# Knowledge of cervical cancer and Pap smear among Uyghur women from Xinjiang, China

**DOI:** 10.1186/s12905-018-0512-5

**Published:** 2018-01-17

**Authors:** Guzhalinuer Abulizi, Tangnuer Abulimiti, Hua Li, Guzhalinuer Abuduxikuer, Patiman Mijiti, Su-Qin Zhang, Ayinuer Maimaiti, Muyasier Tuergan, Ayiguli Simayi, Miherinisha Maimaiti

**Affiliations:** 1 5th Department of Gynecology, Affiliated Tumor Hospital of Xinjiang Medical University, No.789, Suzhou East Road, Urumqi, Xinjiang Uyghur Autonomous Region 830054 China; 2 Surgery Department of Gynecological Oncology, Cancer Center of Guangzhou Medical University, No.78 Heng Ji Gang Road, Guangzhou, 510095 China; 3 6th Department of Gynecology, Affiliated Tumor Hospital of Xinjiang Medical University, No.789, Suzhou East Road, Urumqi, Xinjiang Uyghur Autonomous Region 830054 China; 4 Department of Gynecology, Kelamayi Central Hospital, No.67 Zhun Ge Er Road, Kelamayi, Xinjiang Uyghur Autonomous Region 834000 China; 5 Department of Gynecology, People’s Hospital of Xinjiang Uyghur Autonomous Region, No. 91 Tian Chi Road, Urumqi, Xinjiang Uyghur Autonomous Region 834000 China

## Abstract

**Background:**

Cervical cancer is a significant public health issue in Xinjiang China. In order to provide scientific basis for cervical cancer intervention in Xinjiang, women’s knowledge of cervical cancer was investigated in this study. Besides, relations between Uyghur women’s awareness and their age, educational background, yearly household were evaluated.

**Methods:**

Questionnaire survey was conducted to 7100 Uyghur women from Karkax Hotan and Payzivat Kashgar during 2008 and 2009. Women aged 21 to 70 years, had sexual activity, no history of cervical lesion or cervical cancer were considered to be eligible to the study. Information include participants’ socio-demographic background, personal data, awareness about Pap smear, about cervical cancer and HPV, sources of information acquisition was investigated.

**Results:**

65.1% of the 7100 respondents with primary education level, and 95.0% participants were farmers. Only 7.4% had undertaken Pap smears before, not aware of the importance of the test (97.4% of 7100) was the main reason for not performing Pap smears. 29.3% of total participants had heard about cervical cancer, and only 0.14% (10 out of 7100) had heard about HPV. Top three route of knowledge acquire were television advertises (39.1%), neighbors (21.0%) and health care providers (15.0%). Women younger than 40 years, with higher educational levels and higher income had better awareness of cervical cancer and more willing to accept regular Pap smears.

**Conclusions:**

Uyghur women in Xinjiang had poor knowledge of cervical cancer and HPV infection. Low awareness of women was associated with less household income and lower educational levels. TV shows and education from health care providers may increase women’s participation in cervical cancer control and prevention.

**Electronic supplementary material:**

The online version of this article (doi: 10.1186/s12905-018-0512-5) contains supplementary material, which is available to authorized users.

## Background

Cervical cancer is the most common genital tract malignancy in women and is the second most common cancer in women after breast cancer, with an estimated 530,000 new cases diagnosed each year [1]. During the past 4 decades, cervical cancer incidence and mortality have declined significantly, primarily in western countries, because of the widespread use of the Papanicolaou (Pap) test to screen for cervical abnormalities. Indeed, the rate of decline in cervical cancer incidence and mortality seems to have decreased and has now reached a plateau. However, high incidences of cervical cancer are still being observed and remain a significant problem in developing countries and resource-insufficient areas such as Africa, Asia and Central and South America [2–5].

China is one of the Asian countries with high cervical cancer incidence and mortality rates. According to recent data from a network of 10 Chinese cancer registries, the cervical cancer incidence in China is estimated to be below 4/100,000 [1, 4]. Over the past 30 years, cervical cancer incidence and mortality have decreased steadily in China due to the development of the Pap test and the implementation of screening programs [6]. However, in some remote and poverty-stricken areas, including the Xinjiang Uyghur Autonomous Region, cervical cancer incidence and mortality remain high [7–9].

The Xinjiang Uyghur Autonomous Region is located at the northwest border of China. It is the key transport junction of the ancient Silk Road and is inhabited by people from four world cultural systems with long histories, i.e., China, India, Islam and Rome (Greek). These people have formed a large, mixed community of various nationalities, including Uyghur, Han, Kazak, Hui, Kyrgyz, Mongol, Xibe, Manchu, Uzbek and Russian in Xinjiang [10]. Their economic and, cultural backgrounds play an important role in every detail of Uyghurs’ lives, including marriage, birth, life habits, attitudes and health consciousness [11]. Reports from 2007 indicated that healthcare accessibility in west China was insufficient due to geographical conditions and economic underdevelopment. Xinjiang is mainly a mountainous area, and the population is dispersed over wide areas, making travel inconvenient. Poverty is also a factor restricting the effective utilization of healthcare services. However, the implementation of a New Rural Co-operative Medical System in 2011 has resulted in dramatic improvements in farmers’ medical care. Now, subsidies from state revenue to the west have reached 80%, and the coverage of social security programs for the agricultural population has reached 96.78% [12, 13].

Estimates have shown that the prevalence of cervical cancer among Uyghur minorities is 459/100,000–590/100,000, and the cervical cancer mortality rate is 17.78/100,000. These values are clearly higher than those for the Han, Kazak, Mongol, and Kyrgyz, who also live in Xinjiang. Uyghur cervical cancer patients are younger than patients from other nations. The cervical cancer mortality rate among the Uyghur is the highest of all minority groups in China [7, 8, 14, 15]. In previous studies, a questionnaire was used to survey Xinjiang Uyghur women with cervical cancer, and these studies showed that these women have poor knowledge of cervical cancer and the Pap smear test; many of the surveyed women had never undergone a gynecological examination, and HPV was completely unknown to them [16, 17]. These factors most likely resulted in the steady, high incidence of cervical cancer in Uyghur women [18]. Therefore, cervical cancer is an extremely important public health issue, and reducing the incidence and mortality is urgently required.

The lack of knowledge regarding cancer screening may be a reflection of general poor health education in China. Therefore, comprehensive health education programs are more likely to be beneficial in tackling this problem than disease-specific programs. While numerous studies have conducted population-based analyses of cervical cancer and HPV awareness and knowledge [19–25], virtually no research has been performed exclusively among Uyghur women. In the present study, we examined cervical cancer knowledge and its relationship to educational background and yearly household income among Uyghur women in the Hotan and Kashgar regions of Xinjiang, China, to provide a basis for an educational intervention targeting cervical cancer in Xinjiang.

## Methods

### Samples and data collection

We conducted a cross-sectional study of Uyghur women in the cities of Hotan and Kashgar in 2008 (May to September) and 2009 (June to August) to evaluate their cervical cancer knowledge, and it had taken 8 months to complete the whole survey. The total number of women aged 15–64 were about 193,000 in Karakax, Hotan and 14,000 in Payzivat, Kashgar based on rough estimated data. A total of 5495 women from Hotan and 2313 women from Kashgar were enrolled and accepted the questionnaire survey. Of these, 5000 and 2100 qualified questionnaires were collected. The response rate was 91.0 and 90.8%, respectively. All participants were approached at their homes by trained recruiters, including 4 physicians and 5 healthcare workers. The inclusion criteria were Uyghur women aged 21 to 70 years with a history of sexual intercourse and no diagnosis of cervical cancer or cervical lesions.

The questionnaire was designed in Chinese (Additional file 1) and was translated into Uyghur by the doctors who performed the interview, and the interview was performed during regular, routine primary care home visits.The questionnaire was divided into the following six sections: socio-demographic background, respondents’ personal data, Pap smear knowledge, cervical cancer awareness, HPV awareness, sources of cervical cancer and HPV information, and reasons for not undergoing Pap smears. This study was approved by the Ethics Committee of Xinjiang Medical University, and every participant provided their written informed consent in the Uyghur language to participate in this study. The ethics committee approved this consent procedure.

#### Demographic information

Each subject was asked demographic questions to collect information about her age, education, occupation, monthly household income, marital status, and education level as well as the number of pregnancies and number of children. Sexual history information was obtained, including age at first sexual intercourse.

#### Knowledge and attitude toward pap smears

Participants were asked whether they had ever had a Pap smear and why.

#### Knowledge regarding cervical cancer

Participants were asked whether they had ever heard of cervical cancer and the causes, signs and screening techniques for cervical cancer. Pap smear frequency and cervical cancer prevention knowledge were also obtained.

### Statistical analysis

Data processing and statistical analyses were performed using SPSS 17.0 (IBM Corp, Armonk, NY, USA). Basic descriptive statistics and frequencies were analyzed for all variables. Differences in awareness of cervical cancer by educational level and yearly household income were compared using the χ2 test, and statistical significance was defined as *P* ≤ 0.05.

## Results

### Demographic information

Five thousand four hundred ninety five women from Karasay Hotan and 2313 women from Kezilboy Kashgar were recruited to the study, qualified questionnaire was ontained from 5000 and 2100 women, respectively, and the response rate was 91.0 and 90.8% for Hotan and Kashgar. Total of 7100 women were enroll to this study with a mean age of 42.4 years (21–70 years). The education statuses of the respondents were as follows: 11.3% illiterate, 65.1% elementary school education, 21.1% middle school education and 2.5% college and higher education. The majority of respondents were peasants (95.0%), and the remainder were government employees 2.4% (169), workers 1.4% (98), and other 1.2% (88). The yearly household income of <Ұ5000($758), Ұ5000($758)–10,000($1516), Ұ10,000($1516-)–30,000($4548), Ұ30,000($4548-)–100,000($15,159) and unable to estimate were 68.4% (4858), 24.7% (1755), 2.5% (174), 1.0% (73) and 3.4% (240), respectively.

### Knowledge and attitudes toward pap smears

**Fig. 1:**
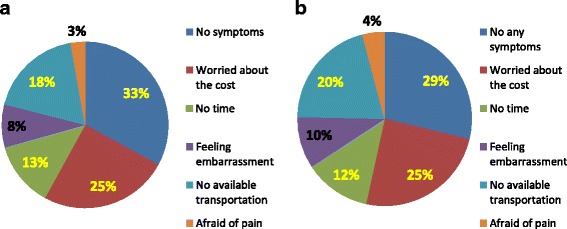
Reasons for not having Pap smears of Uyghur women. In this figure, different reasons for not having regular Pap smears of Uyghur women were illustrated with proportion chart. Reasons were illustrated seperately while women were aware of the significance of pap smears and no aware of significance of pap smears. **a** Reasons for not having regular smears who were unaware of significance (*N*=4126). **b** Reasons for not having regular smears who were aware of significance (*N*=73)

Among the 7100 participants, 97.4% did not know the importance of regular Pap smears. In total, 528 (7.4%) respondents had a previous Pap smear. Among these respondents, more than half (51.0%) had Pap smears because of a doctor’s suggestion, 28.0% had Pap smears because of a health problem, and 21.0% had a Pap smear for regular screening. In total, 6572 of the respondents had never received a Pap smear. The reasons for not having regular smears were as follows: unaware of the significance of Pap smears only (36.1%), unaware of the significance and had no symptoms (20.8%), and unaware of the significance and worried about the cost (15.7%). In total, 73 women knew the importance of Pap smears but had not performed the test due to the following reasons: no symptoms (0.3%), worried about the cost (0.3%), no available transportation (0.2%), no time (0.1%), embarrassment (0.1%), and worried about pain (0.6%) (Fig. 1).

### Awareness of cervical cancer

**Fig. 2:**
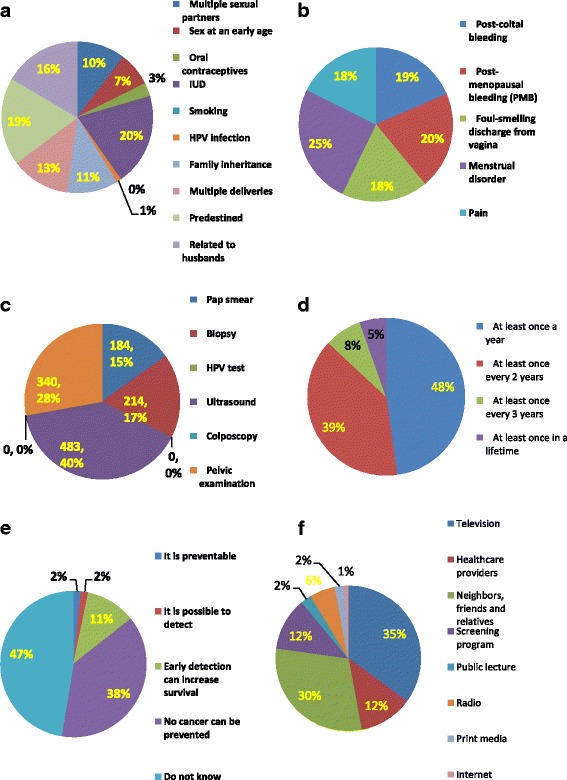
Cervical cancer knowledge of Uyghur women, In this figure, Uyghur women’s awareness about the cause of cervical cancer, sign of cervical cancer, screening method, frequency of doing Pap smears, prevention about cervical cancer and the source of information acquisition were illustrated with proportion chart. **a** What is the cause of cervical cancer? (*N*=278). **b** What is the sign of cervical cancer? (*N*=1772). **c** What is the primary screening method of cervical cancer? (*N*=1221). **d** How often should a Pap smear be performed? (*N*=184). **e** What do you think of cervical cancer prevention? (*N*=7100). **f** Sources of information about cervical cancer (*N*=2082)

Among the study participants, 29.3% had heard of cervical cancer, and only 10 (0.1%) women had heard of HPV (human papillomavirus). The majority of women (96.1%) did not know the causes of cervical cancer. Of the 278 women who knew the causes of cervical cancer, 19.8% believed IUD was a major cause of cervical cancer, 18.7% thought cancer was predestined, and 16.6% believed cervical cancer was related to husbands. Only 1.1% of women recognized HPV infection as a definite pathogenesis of cervical cancer. The signs of cervical cancer were known to 25.0% of respondents, and the percentage of respondents who believed the signs of cervical cancer were post-coital bleeding, post-menopausal bleeding, foul-smelling discharge, menstrual disorder, and pain was 18.6%, 20.5%, 18.2%, 25.2%, 17.6%, respectively. Overall, 39.6% respondents thought an ultrasound could screen for cervical cancer, while 27.8 and 15.1% of women identified pelvic examination and direct biopsy as the main screening methods. In addition, 15.1% of participants believed Pap smears were the primary test of cervical cancer screening, while no one identified HPV as a screening test. Of 184 participants who knew Pap smears were used to screen for cervical cancer, nearly half (47.8%) thought a Pap smear should be performed once a year, and 5.4% believed the smears should be performed once in one’s lifetime. When the respondents were asked about the prevention of cervical cancer, 38.2% of women stated that cervical cancer could never be prevented, and 47.4% of the women did not know whether cervical cancer could be prevented. Only 1.7% of the women stated that cervical cancer was preventable, 1.6% of the women stated that cervical cancer could be detected, and 11.1% of the women stated that early detection could increase the likelihood of survival (Fig. 2).

### Sources of information about cervical cancer

The percentage of participants who had some knowledge of cervical cancer was 30.0% (2132 cases). The sources of information about cervical cancer, in order from highest to lowest reported frequency, were as follows: television (35.1%); neighbors, friends and relatives (30.1%); healthcare providers (12.0%); screening programs (11.8%); radio (5.6%); public lectures (2.4%); print media (2.1%); and network (0.9%). (Fig. 2).

### Correlations between cervical cancer knowledge and age, educational level and income

**Fig. 3:**
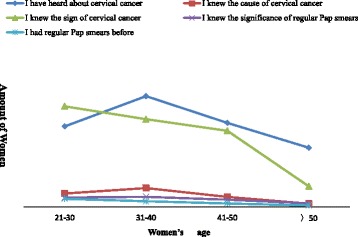
Relationship between women’s knowledge about cervical cancer and their age, In this figure, curve chart was used to descirbe awareness about cervical cancer of women at different age groups. Awareness include women had heard about cervical cancer, who knew the cause and the sign of cervical cancer, knew the significance of Pap smears and had regualr Pap smears before were illustrated

In this study, a women’s cervical cancer knowledge was associated with age. Women who had heard about cervical cancer, who knew the main cause of the disease and who were familiar with the significance of Pap smears were more likely older than 21 years, with a downtrend after 40 years old. Women who knew the symptoms of cervical cancer and who had previously had a Pap smear showed a linear decline after 21 years old (Fig. 3). We aggregated the numbers of women who answered yes to all five questions, compared the summation in different age groups and consulted the curve trend in Fig. 3. We concluded that awareness among Uyghur women was better in the group younger than 40 years (Table 1).

**Table 1:** Women’s knowledge of five items in different age groups

Age group	Yes	No	*χ* ^2^	*P*
≤30 >30	1315 3112	4320 26,753	724.524	0.000
≤40 >40	2763 1664	17,487 13,586	59.525	0.000
≤50 >50	3884 543	27,356 3717	0.338	0.561

Annotation: 5 items: ① had heard about cervical cancer; ②knew the main cause of the disease;③ knew the symptoms of cervical cancer; ④familiar with the significance of Pap smears; ⑤ had received a Pap smear before

In this study, most women had a primary school level of education. The knowledge of cervical cancer in the illiterate group was poor, and almost none of them had ever had a regular check-up or Pap smear. In contrast, women with a college education knew more about cervical cancer, and more women with a college education underwent regular screening. The awareness of cervical cancer was related to education level, as significant differences in cervical cancer knowledge were observed between the different education levels examined (Table 2). Cervical cancer knowledge was also related to higher household income. Women with a higher education level were more aware of cervical cancer and more willing to undergo regular check-ups and Pap smears; however, this difference was not obvious in the high-income group (Table 3).

**Table 2:** Relationship between cervical cancer knowledge and education level

Cervical cancer and HPV knowledge		Education level						
		None (*n* = 803)	Primary (*n* = 4621)	Secondary (*n* = 1498)	College (*n* = 178)	Total (*n* = 7100)	*χ* ^2^	*P*
Heard about cervical cancer	Yes No	63 740	1119 3502	771 727	129 49	2082 5018	751.3	0.000
Knew the cause of cervical cancer	Yes No	4 799	101 4520	105 1393	68 110	278 6822	656.0	0.000
Knew the signs of cervical cancer	Yes No	28 775	870 3751	729 769	145 43	1772 5328	1014.1	0.000
Knew the significance of regular Pap smears	Yes No	3 800	31 4590	18 1480	132 46	184 6916	3706.0	0.000
Had regular Pap smears before	Yes No	2 801	32 4589	23 1475	54 124	111 6989	989.4	0.000

**Table 3:** Relationship between knowledge and yearly household income

Cervical cancer and HPV knowledge		Yearly household income							
		Ұ5000 (*n* = 4858)	Ұ5000–10,000 (*n* = 1755)	Ұ10,000–30,000 (*n* = 174)	Ұ30,000–100,000 (*n* = 73)	Unknown (*n* = 240)	total (*n* = 7100)	χ^2^	*P*
Heard about cervical cancer	Yes	1431	551	53	24	23	2082	49.36	0.000
	No	3427	1204	121	49	217	5018		
Knew the cause of cervical cancer	Yes	160	76	29	8	5	278	92.76	0.000
	No	4698	1679	145	65	235	6822		
Knew the sign of cervical cancer	Yes	1085	559	68	21	39	1772	91.20	0.000
	No	3773	1196	106	52	201	5328		
Knew the significance of regular Pap smears	Yes	90	69	21	3	1	184	90.07	0.000
	No	4768	1686	153	70	239	6916		
Had regular Pap smears before	Yes	51	32	22	5	1	111	163.2	0.000
	No	4807	1723	152	68	239	6989	1	

## Discussion

The prevalence of cervical cancer among Uyghur women in China has remained high over the last 30 years. From 1976 to 2004, the prevalence of cervical cancer among Uyghur women was 590/100000, 459/100000 and 527/100000 [7, 8]. Because cervical cancer can be prevented by early detection and treatment, these rates are alarming and unacceptable. The excess mortality observed in Uyghur women is due in part to low Pap smear screening rates [7, 8]. Disparities in cervical cancer screening and outcomes are influenced by individual factors, including cultural beliefs, customs and habits, linguistic barriers, and socioeconomic status [11].

This study revealed some typical characteristics of Uyghur women living in rural areas. Most Uyghur women have a primary education and work as peasants with a low income. Older age, poverty and a low level of education were correlated with lack of awareness of cervical cancer and all other medical information. Of 7100 women, 2.59% knew the purpose of regular Pap tests. Many women had a Pap smear based on a doctor’s recommendation. This suggests that the majority of women did not know Pap smears were an available method for cervical cancer screening. The most common reasons for not undergoing Pap smears were no awareness regarding the importance, no symptoms and no money. Embarrassment, unavailability of transportation and fear of pain were other reasons that women did not undergo screening. In the two counties studied here, primitive medical facilities and a lack of knowledge among health providers regarding cervical cancer were the most likely factors impeding the participation of local women in screenings. Other cultural barriers may lead to negative opinions about screenings, including concern about exposing private body parts [26]. When asked about their knowledge of cervical cancer, 6822 (96. 08%) women did not know the cause, 5328 women did not know the signs, and 5879 did not know any screening methods. Furthermore, only 7 women had heard about HPV, and only 3 knew that HPV was the cause of cervical cancer. These results show the grave lack of knowledge among Uyghur women regarding cervical cancer, and this lack of knowledge is associated with the high cervical cancer incidence and mortality in this population. Intra Uterine Devices and destiny were considered to be the main causes of cervical cancer. These beliefs may be related to the low educational level of the women. Some women believed that ultrasound pelvic examination could detect cervical cancer, and far fewer women knew that Pap smears and HPV tests were the primary screening methods. Local doctors and other health care providers are partly responsible for this misconception. Watching television was the respondents’ favorite pastime, and television is their best source for cervical cancer knowledge. This finding suggests that a greater number of health educational TV programs or advertisements should be produced in the future to enhance cervical cancer awareness. Statistically significant relationships between education level, cervical cancer knowledge and sex-related factors were found, and cervical cancer knowledge was related to household income.

Women have reported a need for information regarding the indications, benefits, and procedures of cervical cancer screening. Such information is effective at increasing primary screening rates [27]. The factors that reduce participation in cervical screening programs are as follows: poor awareness of the indications and benefits of the cervical smear test, lack of knowledge of cervical cancer and its risk factors, fear of embarrassment and pain, poor understanding of cervical screening methods, and the need for additional information [28].

Little is known regarding the awareness of the risks of cervical cancer among Uyghur women. Our findings seem to coincide with the findings of several national surveys, which suggest that poor, less educated populations are less likely to use cervical cancer screening services [26, 29, 30].

Compared to several studies that assessed HPV awareness, the Uyghur women in our study had absolutely no awareness of HPV. Compared to the Klug et al. meta-analysis, this awareness was low [20]. Numerous studies have found that awareness of HPV is extremely low among minorities, adolescents, and low-income groups [31, 32].

In conclusion, age older than 40 years, poverty and low educational levels are the key factors resulting in poor cervical cancer knowledge among Uyghur women, particularly those who live in the remote countryside. This study showed that several demographic, awareness and attitudinal factors are associated with a decreased likelihood of women utilizing cervical cancer screening. Although these factors are important to consider and policies can address all of them, resources that specifically target the factor most closely associated with uptake, namely, women’s lack of knowledge regarding cervical cancer and its prevention, should be the primary focus. Eliminating this barrier is paramount to achieving the goal of reducing cervical cancer incidence and mortality.

## Conclusions

Uyghur women in China have poor knowledge of cervical cancer and HPV infection, which is associated with low household income and low educational levels. Education via TV and health care providers may improve compliance with programs aimed at cervical cancer prevention and treatment.

## Additional files

Additional file 1: Questionnaire 1. Questionnaire in Chinese adopted in the survey Original questionnaire was provided in this file which was adopted in this reasearch. (PDF 321 kb)
