# Supplementary material for: Knowledge of cervical cancer and Pap smear among Uyghur women from Xinjiang, China
Source: BMC Womens Health. 2018 Jan 17;18:21. doi: 10.1186/s12905-018-0512-5 (PMC5773149; doi:10.1186/s12905-018-0512-5)
Supplement: Supplementary file 1 — Questionnaire in Chinese adopted in the survey Original questionnaire was provided in this file which was adopted in this reasearch. (PDF 321 kb) [file 12905_2018_512_MOESM1_ESM.pdf]

宫颈癌相关知识调查问卷

调查对象姓名 ： ________________　　　　**调查对象 ID 号：**|__|__|__|__|__|－|__|__|__|__|

身份证号： |__|__|__|__|__|__|__|__|__|__|__|__|__|__|__|__|__|__|

年龄：|__|__|

永久住址： ____

<u>1.一般情况、婚姻生育状况、卫生情况</u>

1.1.1 婚姻：

□①已婚　□②离婚　□③再婚

□④丧偶

1.1.2 文化程度：

□①无　□②小学　□③中学

□④大专　□⑤本科　□⑥研究生

1.1.3 职业：

□①农民　□②家属　□③个体户

□④干部　□⑤工人

1.1.4 爱人的文化程度：

□①无　□②小学　□③中学

□④大专　□⑤本科　□⑥研究生

1.1.5 爱人的职业：

□①农民　□②家属　□③个体户

□④干部　□⑤工人

1.1.6 家庭年收入：

□①＜5 千元　□②5 千-1 万元□③1 万-3 万元

□④3-10 万元 □⑤＞10 万元　□⑥不愿意透露

1.2.1 家族有无肿瘤家族史？

□①有　□②无(跳至问题 1.3.1)

1.2.2 如有为何种肿瘤：（可多选）

□①宫颈癌　□②食管癌　□③口腔癌

□④肛门癌　□⑤其他（说明________）

1.2.3 患者与自己的关系（可多选）

□①母亲　□②姐妹　□③姥姥

□④奶奶　□⑤姑姑　□⑥姨姨

□⑦表姐妹　□⑧堂姐妹　□⑨侄女

1.3.1 结婚年龄：　|__|__|岁

1.3.2 初次分娩年龄：　|__|__|岁

1.3.3 怀孕次数：　|__|__|次

1.3.4 分娩次数：　|__|__|次

1.3.5 分娩方式：（可多选）

□①阴道分娩顺产　□②阴道分娩难产

□③剖腹产

1.3.6 分娩地点：

□①家　□②医院　□③两者均有

1.4.1 结婚次数：　|__|__|次

1.4.2 爱人的结婚次数：　　　　　|__|__|次

1.4.3 同房次数：

☐①≤1 次/周　☐②2 次/周　☐③3 次/周

☐④≥4 次/周　☐⑤1 次/2 周　☐⑥1 次/3 周

☐⑦≥1 次/4 周

1.4.4 是否愿意避孕：

☐①是　　　☐②否

1.4.5 避孕方法：（可多选）

☐①宫内节育器 ☐②体外避孕 ☐③口服避孕药

☐④避孕套　　☐⑤输卵管结扎

☐⑥未避孕　　☐⑦绝经　　☐⑧其他（说明__）

1.4.6 对避孕套的态度：

☐①可以　　☐②一般　　☐③烦感

☐④喜欢　　☐⑤未使用过

1.5.1 月经期用材：（可多选）

☐①卫生纸　　☐②其他纸　　☐③布

☐④卫生巾

1.5.2 厕所用材：（可多选）

☐①卫生纸　　☐②其他纸　☐③布

☐④土块

1.5.3 同房前洗外阴：

☐①是　　　☐②否

1.5.4 洗外阴前洗手：

☐①直接洗　　☐②用肥皂

1.5.5 同房前洗阴道：

☐①是　　　☐②否

1.5.6 同房后洗外阴：

☐①是　　　☐②否

1.5.7 同房后洗阴道：

☐①是　　　☐②否

1.5.8 做礼拜前洗阴道：

☐①是　　　☐②否

1.6.1 同房后洗澡：

☐①是　　　☐②否

1.6.2 洗澡频率：

☐① 1-3 天/次 ☐②3-5 天/次☐③7 天/次

☐④14 天/次　☐⑤1 月/次　☐⑥ 3 月/次

☐⑦1 年/2 次

1.6.3 更换内裤频率：

☐①1-3 天/次　☐②3-5 天/次　☐③7 天/次

☐④14 天/次　☐⑤1 月/次

1.6.4 每次礼拜前是否更换垫布

☐①是　　　☐②否

2.选择医院

2.1 当出现身体不适首先选择：

□①维医　　　□②中医　　　□③西医

2.2 当被查出疾病时首先选择：

□①维医保守治疗

□②中医保守治疗

□③西医手术治疗

3.对宫颈癌与 HPV 认知程度

3.1 对定期检查和对巴氏涂片认知程度

3.1.1 做定期检查的意义 ：

□①知道　　　□②不知道

3.1.2 定期行涂片的意义 ：

□①知道　　　□②不知道

3.1.3 曾经是否做过妇科检查：

□①是　　　　□②否(跳至问题 3.1.5)

3.1.4 曾经做过妇科检查的原因：

□①因为有症状□②常规体检

□③认为妇检可以解决所有的妇科问题

□④医生建议　□⑤有免费体检项目

3.1.5 曾经是否做过宫颈涂片：

□①是　　　　□②否(跳至问题 3.2)

3.1.6 曾经做过宫颈涂片的原因：

□①常规体检　□②因为有症状 □③医生建议

3.2 妇女对宫颈癌及其相关知识的认知程度

3.2.1 是否知道宫颈癌：

□①是　　　　□②否 （跳至问题 3.2.8）

3.2.2 引起宫颈癌的高危因素：（可多选）

□①多个性伴侣 □②性生活过早

□③宫内节育器 □④口服避孕药 □⑤HPV

□⑥宫颈癌家族史　　　　　　□⑦多胎

□⑧命中注定　□⑨跟丈夫有关 □⑩不知道

3.2.3 宫颈癌的症状： （可多选）

□①同房后出血 □②绝经后阴道流血

□③阴道排出腥臭味分泌物

□④月经紊乱　□⑤疼痛　　　□⑥不知道

3.2.4 知道宫颈癌的好发年龄:

□①是　　　　□②否

3.2.5 宫颈癌筛查的方法有哪些：（可多选）

□①液基细胞学 □②宫颈活检　□③超声检查

□④妇检（双合诊检查）　　　□⑤HPV 检□⑥不知道

3.2.6 宫颈癌筛查的时间间隔多长为合适：

□①至少每年一次

□②至少两年一次

□③至少每三年一次

☐④至少终身一次　　　　☐⑤不知道

3.2.7 对宫颈癌的预防了解情况：

☐①可以预防　　　　☐②可以在癌前期发现

☐③可以早期发现　　　　☐④癌症无法防治

☐⑤不知道

3.2.8 不参加定期普查及宫颈涂片的原因：（可多选）

☐①不知道其意义　☐②没有任何症状

☐③没有时间　☐④没有钱

☐⑤交通不方便　☐⑥非常尴尬

☐⑦爱人不同意　☐⑧宗教原因

☐⑨害怕疼痛

3.3 妇女对 HPV 的认知程度

3.3.1 知道什么是 HPV：

☐①是　☐②否

3.3.2 任何关于 HPV 的信息：

☐①知道　☐②不知道（跳至问题 4.1）

3.3.3HPV 是宫颈癌的重要病因：

☐①知道　☐②不知道

3.3.4HPV 可以引起男女生殖器疣：

☐ ①知道　☐②不知道

3.3.5HPV 可以通过性传播：

☐①知道　☐②不知道

3.3.6HPV 和宫颈癌相关知识的来源：

☐①电视　☐②收音机

☐③印刷产品　☐④亲戚和朋友

☐⑤清真寺　☐⑥网络

☐⑦广告　☐⑧集体讲座

☐⑨曾参加过普查项目

☐⑩医院和医护人员

4.配偶个人史

4.1 丈夫是否吸烟：

☐①是　☐②否（跳至问题 5.6）

4.2 丈夫吸烟频率：　|__|__|支/天(香烟)

　　　　　　　　|__|__|克/天（莫合烟）

4.3丈夫吸烟地点：

☐①屋内　☐②院内　☐③院外

4.4丈夫开始吸烟年龄: |__|__|岁

4.5 丈夫是否已戒烟：

☐①是　☐②否

4.6丈夫是否吸毒：

☐①是　☐②否（跳至问题5.8）

4.7 丈夫如吸毒为下列哪种：（可多选）

☐①大麻　☐②海洛因　☐③吗啡

调查员签字：______________________________ 日期：|__|__|__|__|年|__|__|月|__|__|日
